# Supplementary material for: Magnetic skyrmion logic gates: conversion, duplication and merging of skyrmions
Source: Sci Rep. 2015 Mar 24;5:9400. doi: 10.1038/srep09400 (PMC4371840; doi:10.1038/srep09400)
Supplement: Supplementary Information [file srep09400-s1.pdf]

Magnetic skyrmion logic gates: conversion, duplication and merging of skyrmions

Xichao Zhang[1], Motohiko Ezawa[2, *], Yan Zhou[1, 3, †]

1. Department of Physics, University of Hong Kong, Hong Kong, China

2. Department of Applied Physics, University of Tokyo, Hongo 7-3-1, Tokyo 113-8656, Japan

3. Center of Theoretical and Computational Physics, University of Hong Kong, Hong Kong, China

***E-mail: ezawa@ap.t.u-tokyo.ac.jp**
†E-mail: yanzhou@hku.hk

Supplementary Note 1: Typical examples of skyrmions, merons and bimerons

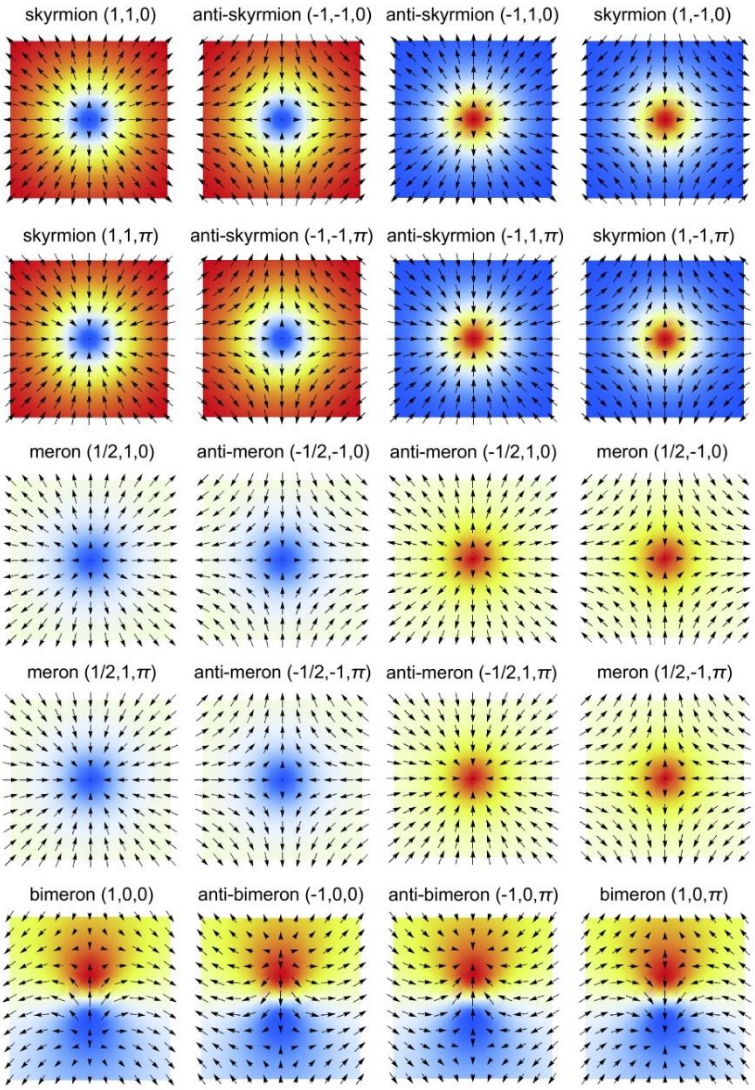

Supplementary Figure 1. Typical examples of skyrmions, merons and bimerons. The arrows represent the in-plane magnetization direction; the perpendicular magnetization is color coded. Blue (red) region shows that the *z*-component of spin is negative (positive), while white region shows that the spin direction is in-plane.

Supplementary Note 2: Impact of the width of the channel on skyrmion-conversion geometry

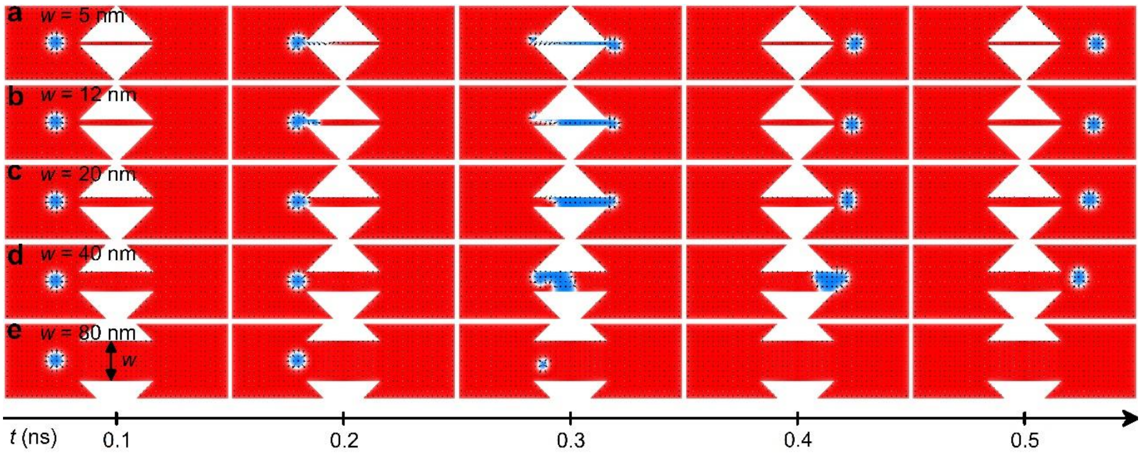

Supplementary Figure 2. Impact of the width of the channel on skyrmion-conversion geometry. The D is 3.5 mJ m^{-2} in the input side and -3.5 mJ m^{-2} in the output side, while a gradient change from 3.5 mJ m^{-2} to -3.5 mJ m^{-2} is set in the narrow channel. The background points +z. A current density of 3×10^{12} A m^{-2} (the value is of the input or output side) is applied along -x for 0 ns < t < 0.5 ns. The width of the input and output sides is 150 nm and the length of the sample is 450 nm. The width of the narrow channel is **a**, 5 nm; **b**, 12 nm; **c**, 20 nm; **d**, 40 nm; **e**, 80 nm.

We have carried out a series of simulations with different widths of the narrow channel corresponding to the case of Fig. 2b in the main text (see Fig. 2b in the main text). As shown in Supplementary Figure 2, with the same parameter setting and profile, the skyrmion in the input side can be successfully converted to a skyrmion with opposite in-going helicity in the output side when the width of the narrow channel is not larger than the skyrmion size. As shown in Supplementary Figure 2e, if the narrow channel width is larger than the skyrmion size, the skyrmion will not convert into a domain wall pair. Instead, the size of the skyrmion will be reduced since the DMI constant decreases from positive value at the left end of the channel to zero at mid of the channel. The skyrmion will ultimately be annihilated in the channel without enough DMI.

Supplementary Note 3: Impact of the opening angle of the Y-junction

We have also studied the impact of the opening angle of the fan-out Y-junction channel in the skyrmion duplication/merging geometry. The results are shown in Supplementary Figure 3. It can be seen that the domain wall pair can be split into two domain wall pairs at the fan-out Y-junction geometries with an opening angle of 37 degrees, 53 degrees and 90 degrees. However, it should be noted that if the opening angle is closed to/equal to 180 degrees, it can be expected that the domain wall is hard to be split into two domain wall at low current density of driving current. A very abrupt change of the domain wall propagation direction may also lead to destroy of the domain wall. On the other hand, from the point view of experiment, an opening angle smaller than 30 degrees may be hard to be constructed. Thus, an opening angle around 60 degrees could be a trade-off.

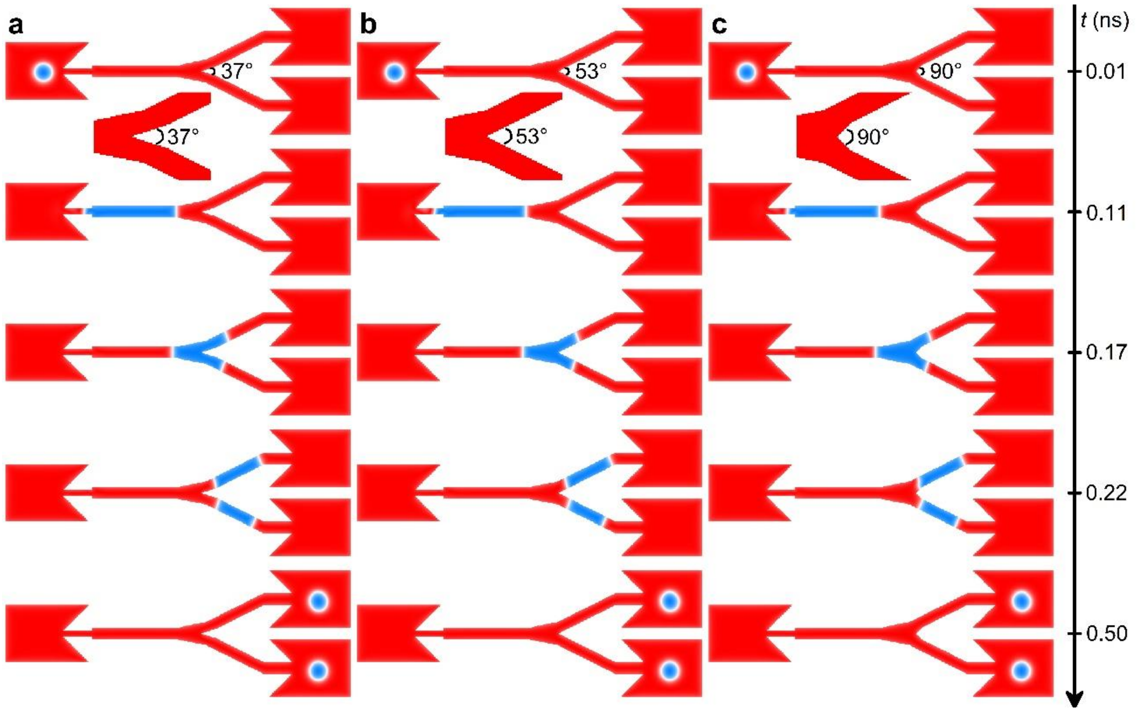

Supplementary Figure 3. Impact of the opening angle of the fan-out Y-junction. The *D* is 3.5 mJ m^{-2}. The initial background magnetization of the sample points +*z*. A current density of 5×10^{12} A m^{-2} (the value is of the input side) is applied along -*x* direction for 0 ns < *t* < 0.50 ns. The opening angle of the fan-out Y-junction is **a**, 37 degrees; **b**, 53 degrees; **c**, 90 degrees.

Supplementary Movie Legends:

Supplementary Movie 1. Conversion between a skyrmion and a skyrmion with identical out-going helicity: the *D* in the sample is 3.5 mJ m^{-2}; the background points +*z*; a current density of 3×10^{12} A m^{-2} (the value is of the input or output side, similarly hereinafter) is applied along -*x* for 0 ns < *t* < 0.56 ns followed by a relaxation until *t* = 1 ns.

Supplementary Movie 2. Conversion between a skyrmion and a skyrmion with opposite in-going helicity: the *D* is 3.5 mJ m^{-2} in the input side and -3.5 mJ m^{-2} in the output side, while a gradient transition from 3.5 mJ m^{-2} to -3.5 mJ m^{-2} is set in the narrow channel; the background points +*z*; a current density of 3×10^{12} A m^{-2} is applied along -*x* for 0 ns < *t* < 0.51 ns and then is the relaxation until *t* = 1 ns.

Supplementary Movie 3. Conversion between a skyrmion and an anti-skyrmion with opposite in-going helicity: the *D* in the sample is 3.5 mJ m^{-2}; the background of the input side points +*z*, while it points -*z* in the output side; a current density of 2.67×10^{12} A m^{-2} is applied along -*x* for 0 ns < *t* < 0.51 ns followed by a relaxation until *t* = 1 ns.

Supplementary Movie 4. Conversion between a skyrmion and an anti-skyrmion with identical out-going helicity: the profile of *D* is the same as that in **b** and the profile of background is the same as that in **c**; a current density of 2.67×10^{12} A m^{-2} is applied along -*x*

for 0 ns < t < 0.51 ns followed by a relaxation until t = 1 ns.

Supplementary Movie 5. Conversion between a skyrmion and an anti-bimeron: the D in the sample is 3.5 mJ m^{-2}; the anisotropy K is 0.8 MJ m^{-3} in the input side and -0.8 MJ m^{-3} in the output side, while a gradient transition from 0.8 MJ m^{-3} to -0.8 MJ m^{-3} is set in the narrow channel. The initial background magnetization of the input side points +z, while it is mostly aligned along -x direction in the output side; a current density of 9×10^{12} A m^{-2} (the value is of the input or output side, similarly hereinafter) is applied along -x direction for 0 ns < t < 0.17 ns followed by a relaxation without applying any current until t = 1 ns.

Supplementary Movie 6. Conversion between a skyrmion and a bimeron: the D is 3.5 mJ m^{-2}; the profile of the anisotropy is the same as that in Supplementary Movie 5. The initial background magnetization of the input side points +z, while it is mostly aligned along +x direction in the right output side; a current density of 10×10^{12} A m^{-2} is applied along -x direction for 0 ns < t < 0.15 ns followed by a relaxation until t = 1 ns.

Supplementary Movie 7. Duplication of a skyrmion: the D is 3.5 mJ m^{-2}; the initial background magnetization of the sample points +z; a current density of 5×10^{12} A m^{-2} (the value is of the input side) is applied along -x direction for 0 ns < t < 0.49 ns followed by a relaxation without applying any current until t = 1 ns.

Supplementary Movie 8. Merging of two skyrmions: the D is 3.5 mJ m^{-2}; the initial background magnetization of the sample points +z; a current density of 4×10^{12} A m^{-2} (the value is of the output side) is applied along -x direction for 0 ns < t < 0.64 ns followed by a relaxation until t = 1 ns.

Supplementary Movie 9. The basic operation of OR gate 1 + 0 = 1: there is a skyrmion in the input A and no skyrmion in the input B at initial time, which represents input = 1 + 0; a current density of 7×10^{12} A m^{-2} (the value is of the output side, similarly hereinafter) is applied along -x direction for 0 ns < t < 0.39 ns followed by a relaxation without applying any current until t = 1 ns. At t = 1 ns, a stable skyrmion is in the output side, which represents output = 1.

Supplementary Movie 10. The basic operation of the OR gate 0 + 1 = 1: there is a skyrmion in the input B side and no skyrmion in the input A side at initial time, which represents input = 0 + 1; a current density of 7×10^{12} A m^{-2} is applied along -x direction for 0 ns < t < 0.39 ns followed by a relaxation without applying any current until t = 1 ns. At t = 1 ns, a stable skyrmion is in the output side, which represents output = 1.

Supplementary Movie 11. The basic operation of the OR gate 1 + 1 = 1: there is a skyrmion in both the input A side and the input B side, which represents input = 1 + 1; a current density of 4×10^{12} A m^{-2} is applied along -x direction for 0 ns < t < 0.64 ns followed by a relaxation

without applying any current until t = 1 ns. At t = 1 ns, a stable skyrmion is in the output side, which represents output = 1.

Supplementary Movie 12. The basic operation of AND gate 1 + 0 = 0: there is a skyrmion in the input A side and no skyrmion in the input B side at initial time, which represents input = 1 + 0; a current density of 4×10^{12} A m^{-2} (the value is of the output side, similarly hereinafter) is applied along $-x$ direction for 0 ns < t < 0.81 ns followed by a relaxation without applying any current until t = 1 ns. At t = 1 ns, no skyrmion is in the output side, which represents output = 0.

Supplementary Movie 13. The basic operation of the AND gate 0 + 1 = 0: there is a skyrmion in the input B side and no skyrmion in the input A side at initial time, which represents input = 0 + 1; a current density of 4×10^{12} A m^{-2} is applied along $-x$ direction for 0 ns < t < 0.81 ns followed by a relaxation without applying any current until t = 1 ns. At t = 1 ns, no skyrmion is in the output side, which represents output = 0.

Supplementary Movie 14. The basic operation of the AND gate 1 + 1 = 1: there is a skyrmion in both the input A side and the input B side, which represents input = 1 + 1; a current density of 4×10^{12} A m^{-2} is applied along $-x$ direction for 0 ns < t < 0.81 ns followed by a relaxation without applying any current until t = 1 ns. At t = 1 ns, a stable skyrmion is in the output side, which represents output = 1.
