# Supplementary figures and images for: Magnetic skyrmion logic gates: conversion, duplication and merging of skyrmions
Source: Sci Rep. 2015 Mar 24;5:9400. doi: 10.1038/srep09400 (PMC4371840; doi:10.1038/srep09400)

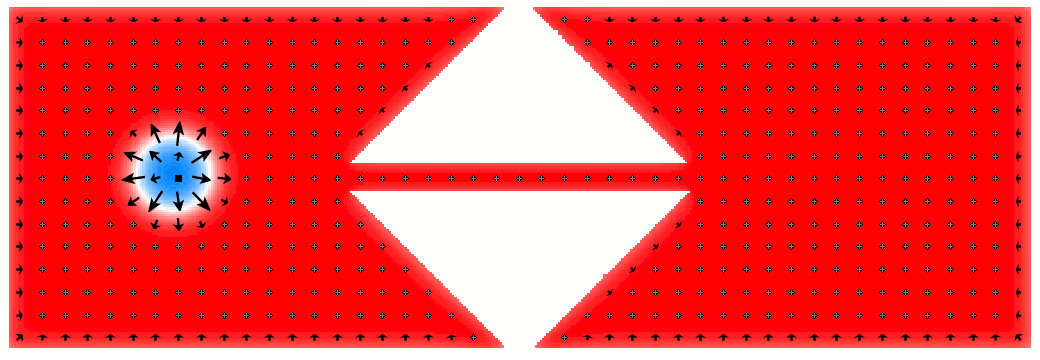

Supplement: Supplementary Information — Supplementary Movie 1 [file srep09400-s2.gif]

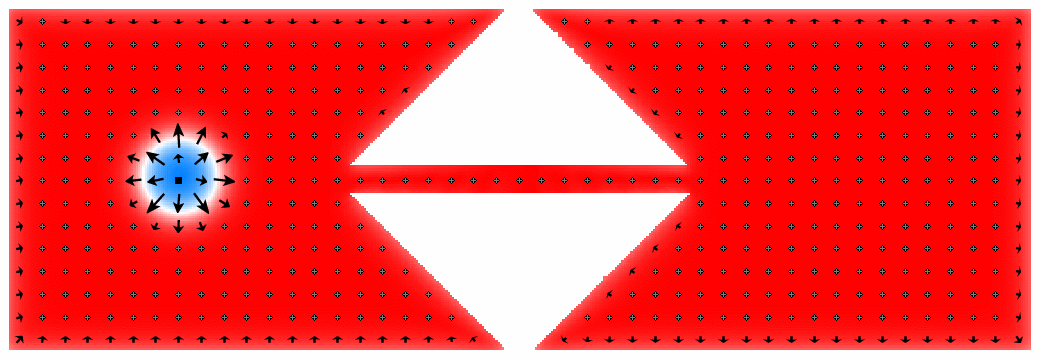

Supplement: Supplementary Information — Supplementary Movie 2 [file srep09400-s3.gif]

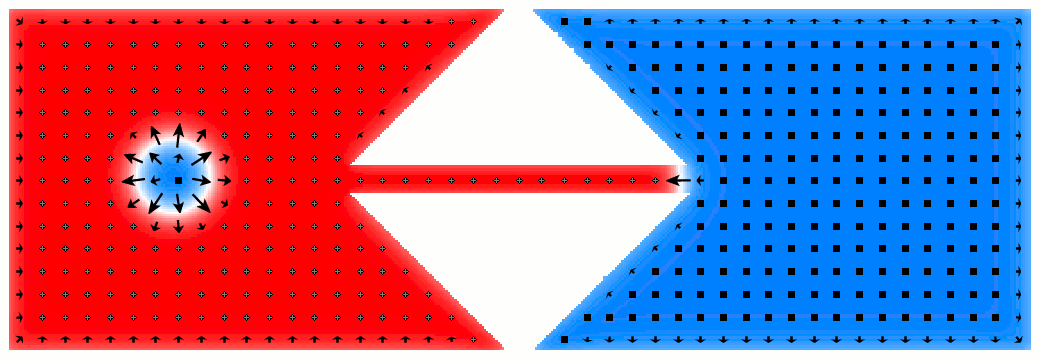

Supplement: Supplementary Information — Supplementary Movie 3 [file srep09400-s4.gif]

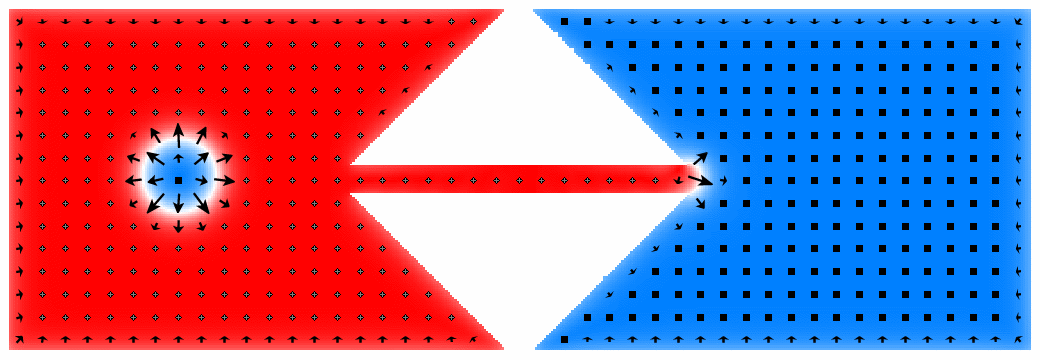

Supplement: Supplementary Information — Supplementary Movie 4 [file srep09400-s5.gif]

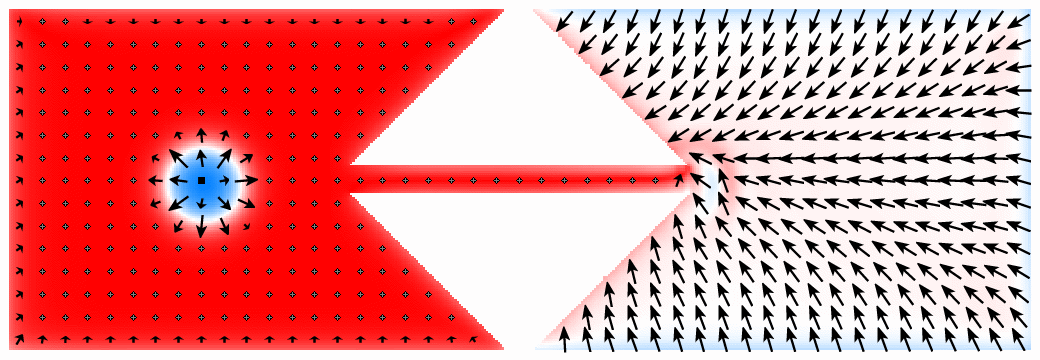

Supplement: Supplementary Information — Supplementary Movie 5 [file srep09400-s6.gif]

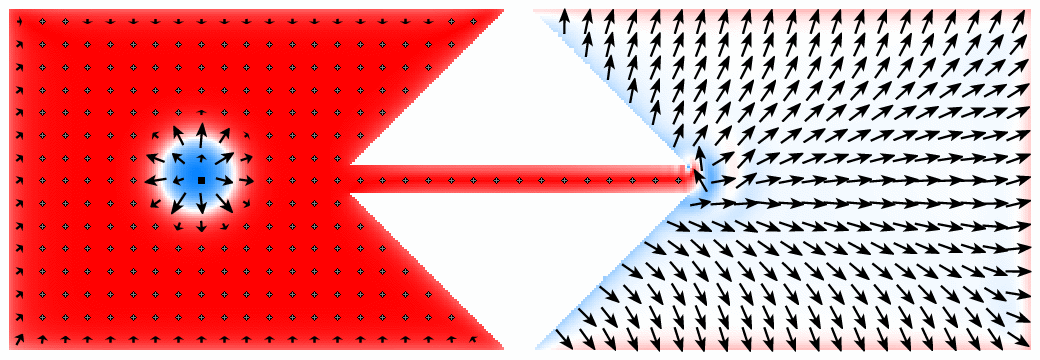

Supplement: Supplementary Information — Supplementary Movie 6 [file srep09400-s7.gif]

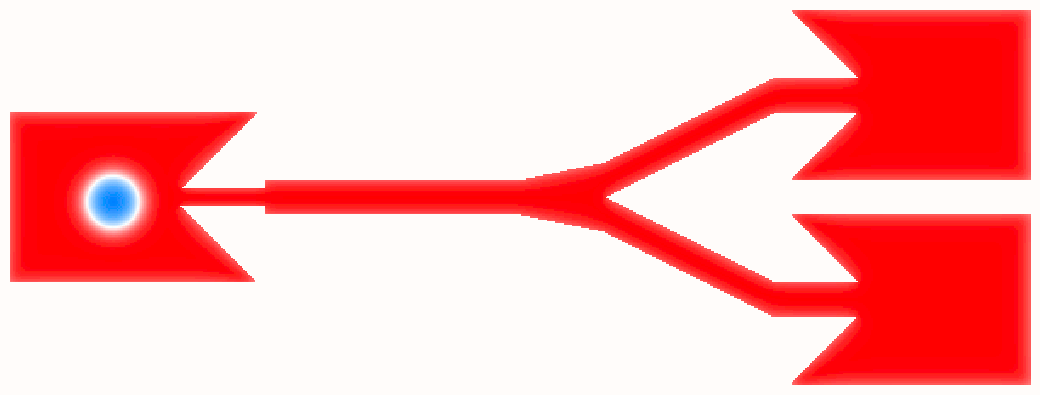

Supplement: Supplementary Information — Supplementary Movie 7 [file srep09400-s8.gif]

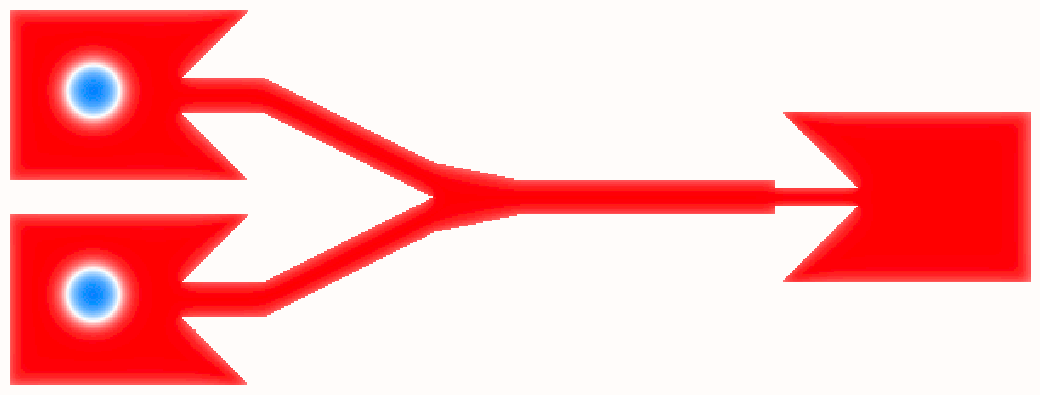

Supplement: Supplementary Information — Supplementary Movie 8 [file srep09400-s9.gif]

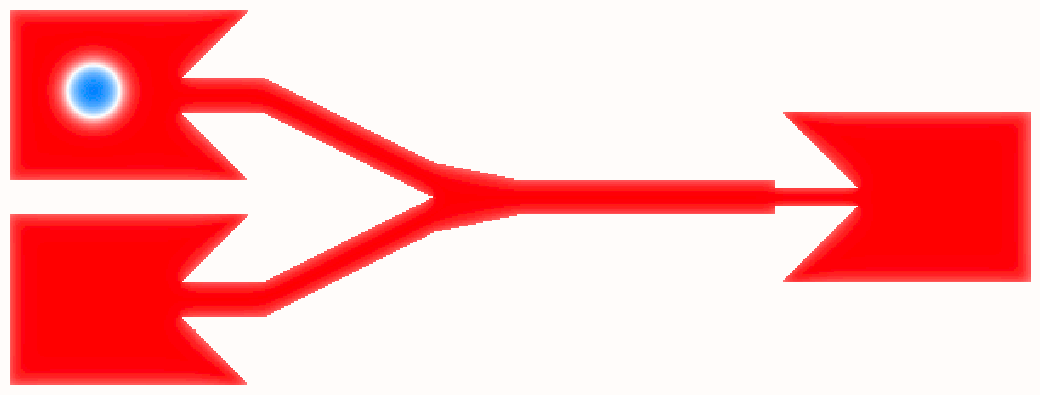

Supplement: Supplementary Information — Supplementary Movie 9 [file srep09400-s10.gif]

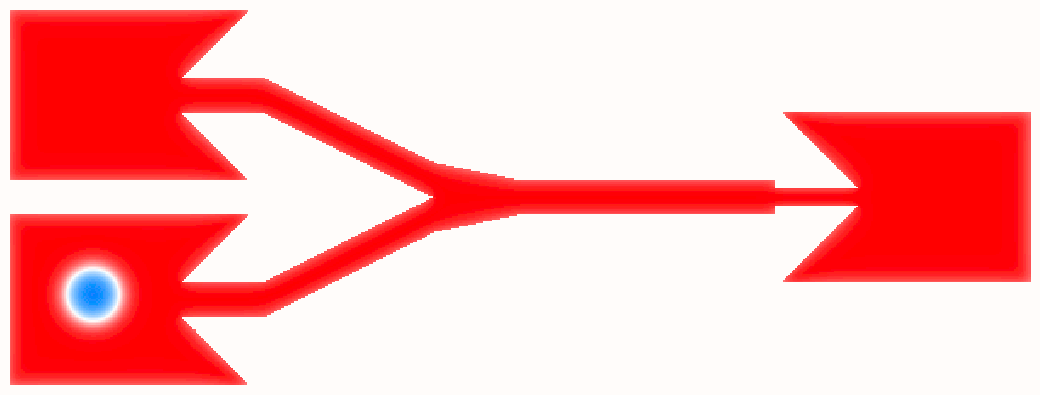

Supplement: Supplementary Information — Supplementary Movie 10 [file srep09400-s11.gif]

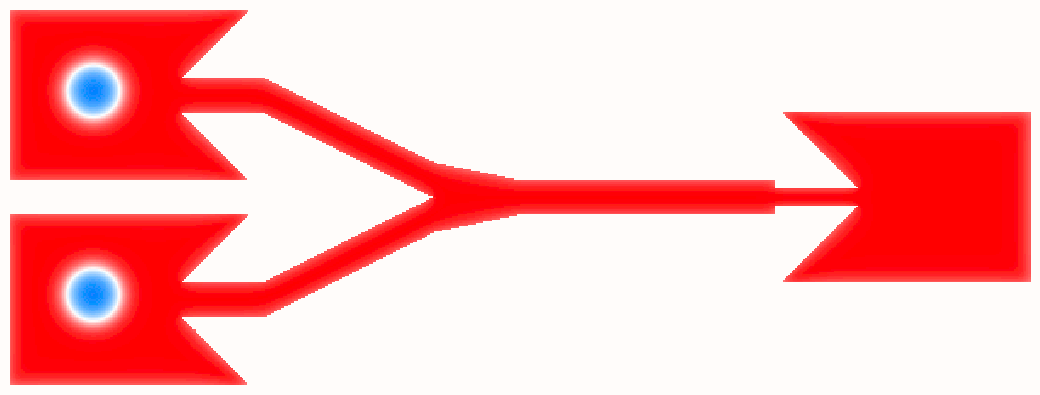

Supplement: Supplementary Information — Supplementary Movie 11 [file srep09400-s12.gif]

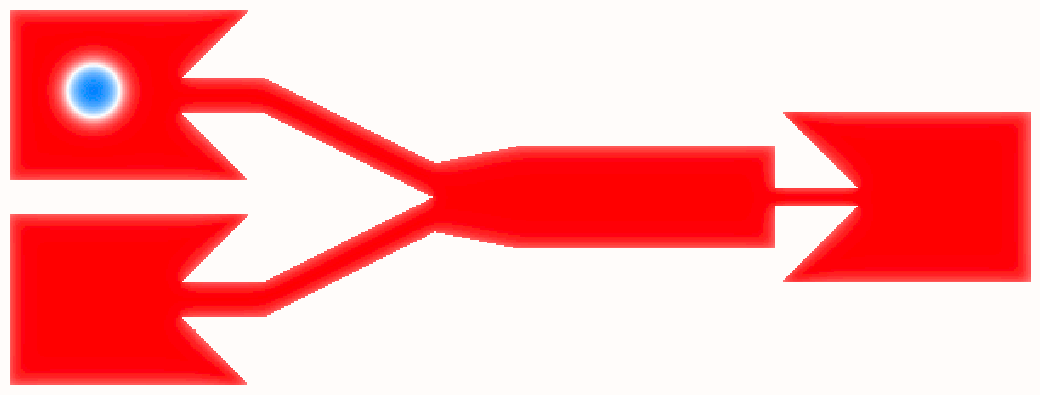

Supplement: Supplementary Information — Supplementary Movie 12 [file srep09400-s13.gif]

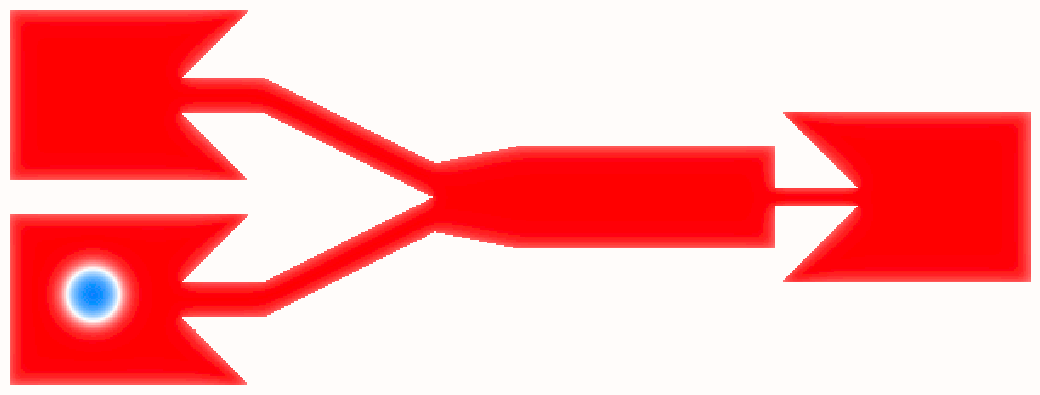

Supplement: Supplementary Information — Supplementary Movie 13 [file srep09400-s14.gif]

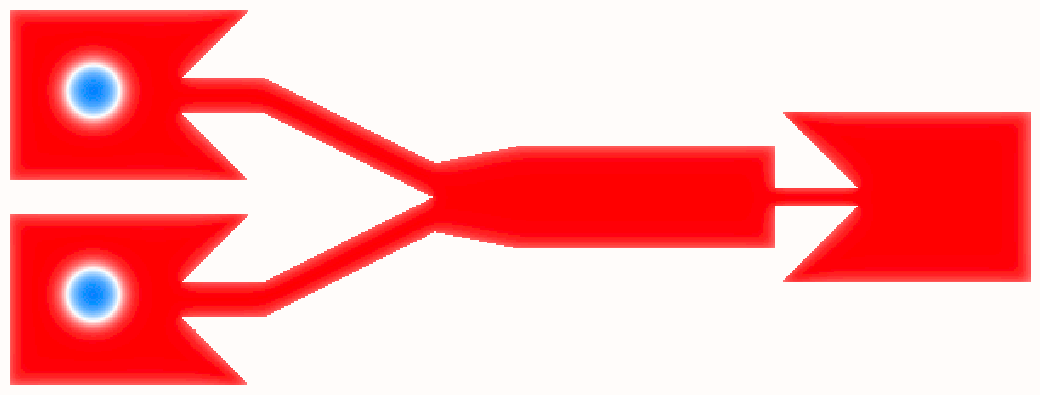

Supplement: Supplementary Information — Supplementary Movie 14 [file srep09400-s15.gif]
